# *STK11/LKB1* Modulation of the Immune Response in Lung Cancer: From Biology to Therapeutic Impact

**DOI:** 10.3390/cells10113129

**Published:** 2021-11-11

**Authors:** Elvire Pons-Tostivint, Alexandre Lugat, Jean-François Fontenau, Marc Guillaume Denis, Jaafar Bennouna

**Affiliations:** 1 Medical Oncology Department, Nantes University Hospital, 44000 Nantes, France; 2 Center for Research in Cancerology and Immunology Nantes-Angers (CRCINA), University of Nantes, INSERM UMR 1232, 44000 Nantes, France; alexandre.lugat@chu-nantes.fr (A.L.); jean-francois.fonteneau@inserm.fr (J.-F.F.); j.bennouna@hopital-foch.com (J.B.); 3 Department of Biochemistry, Nantes University Hospital, 44000 Nantes, France; marc.denis@chu-nantes.fr; 4 Medical Oncology Department, Hopital Foch, 75073 Suresnes, France

**Keywords:** *STK11/LKB1*, non-small cell lung cancer, immunotherapy, biomarker, KRAS

## Abstract

The *STK11/LKB1* gene codes for liver kinase B1 (*STK11/LKB1*), a highly conserved serine/threonine kinase involved in many energy-related cellular processes. The canonical tumor-suppressive role for *STK11/LKB1* involves the activation of AMPK-related kinases, a master regulator of cell survival during stress conditions. In pre-clinical models, inactivation of *STK11/LKB1* leads to the progression of lung cancer with the acquisition of metastatic properties. Moreover, preclinical and clinical data have shown that inactivation of *STK11/LKB1* is associated with an inert tumor immune microenvironment, with a reduced density of infiltrating cytotoxic CD8^+^ T lymphocytes, a lower expression of PD-(L)1, and a neutrophil-enriched tumor microenvironment. In this review, we first describe the biological function of *STK11/LKB1* and the role of its inactivation in cancer cells. We report descriptive epidemiology, co-occurring genomic alterations, and prognostic impact for lung cancer patients. Finally, we discuss recent data based on pre-clinical models and lung cancer cohorts analyzing the results of *STK11/LKB1* alterations on the immune system and response or resistance to immune checkpoint inhibitors.

## 1. Introduction

Germinal heterozygous mutations in the serine/threonine kinase 11 (*STK11)* gene were first identified as the causal mutation of the Peutz-Jeghers Syndrome, an autosomal dominant condition characterized by multiple hamartomatous polyps in the gastrointestinal tract and an increased cancer risk [1]. The *STK11* gene codes for liver kinase B1 (LKB1), a highly conserved serine/threonine kinase that acts as a sensor of cellular energy, giving it a special role in cellular metabolism, especially in cancer cells [2]. Somatic *STK11* alterations have been described in many different tumor types, and they represent the second most altered tumor suppressor gene after *TP53* in non-small cell lung cancer (NSCLC).

Targeted therapies have significantly improved survival in non-squamous NSCLC patients expressing specific molecular alterations such as epidermal growth factor receptor (*EGFR*)-sensitizing mutations, *ALK* and *ROS-1* rearrangements [3]. Outside of this selective context of oncogenic addiction, chemotherapy associated with immune checkpoint inhibitors (ICI) or ICI alone remains the first-line treatment option [3]. Unfortunately, most patients with advanced NSCLC relapse after treatment with PD-(L)1 (programmed death (ligand) 1) axis inhibitors. The landscape of primary and secondary resistance to PD-1 blockade is still largely unknown, while it is currently well known that molecular alterations of tumors are involved in the shaping of their immune microenvironment.

*STK11/LKB1* alterations may have a major impact on the immune microenvironment of lung tumors, and evidence suggests its potential role in the resistance to ICI. This review aims to provide an overview of *STK11/LKB1* biological functions and its implications in carcinogenesis. We present pre-clinical and clinical data evaluating the relevance of *STK11/LKB1* alterations in NSCLC and its correlation with the immune system and cellular processes. Finally, we discuss available data reporting the therapeutic impact of *STK11/LKB1* alterations on ICI efficacy in NSCLC patients.

## 2. *STK11/LKB1*: An Overview of Biological Functions

**Figure 1:**
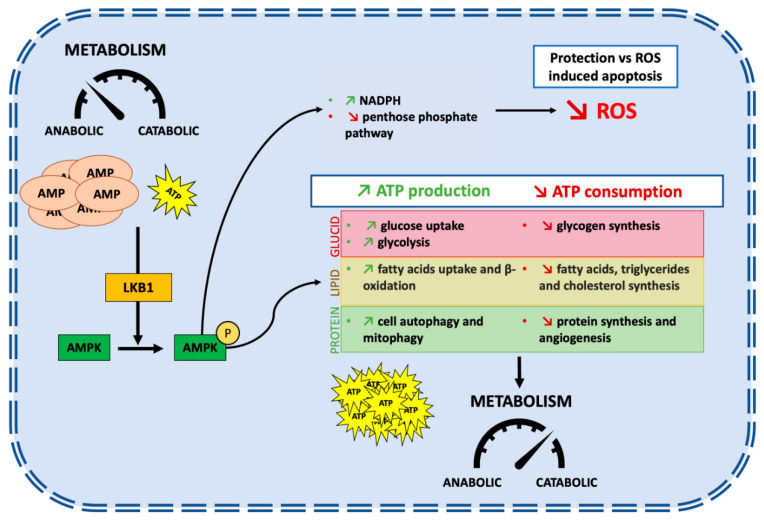
*STK11/LBK1* promotes cell survival under stress conditions by allowing a switch from an anabolic to a catabolic metabolism. Under energy deprivation, the accumulation of AMP and the decreased levels of ATP lead to activation of LKB1 which phosphorylate AMPK at Thr-172 in an ⍺ subunit resulting in its activation. AMPK is involved in multiple metabolic pathways to increase ATP production and to stop ATP consumption switching metabolism from an anabolic state to a catabolic state. This pathway promotes cell survival under stress conditions but impairs cell growth and proliferation.

The *STK11* gene is located at the telomeric region of the short arm of chromosome 19 (19p13.3) [4,5,6]. Nine exons code for a sequence of 443-amino acids which form the LKB1 protein [7]. *STK11/LKB1* is an essential master upstream kinase that activates AMP-activated protein kinase (AMPK) in case of energy deprivation (Figure 1). AMPK is a central metabolic checkpoint in the cell that regulates glucose and lipid metabolism in response to nutrients and energy variations, as well as other cellular functions such as autophagy and polarity. Thus, under nutrient deprivation or hypoxia, an AMP accumulation occurs, in conjunction with ATP depletion, leading to the direct activation of AMPK by *STK11/LKB1* [8]. Activation of AMPK in conjunction with other regulators allows a switch from an anabolic to a catabolic metabolism, promoting cell survival in energy stress conditions [9]. It triggers physiological processes that regenerate ATP, as well as activating 12 other kinases of the AMPK subfamily [10]. AMPK is involved in multiple metabolic ways to increase ATP cellular levels, both by promoting its production and reducing its consumption. Among others, AMPK promotes lipid catabolism by increasing fatty acid uptake and β-oxidation, leading to ATP and NADPH production [11,12,13,14]. LKB1/AMPK is also involved in the glucose catabolism by an increase in glucose uptake and the promotion of the glycolysis process [15,16].

Protein synthesis is energetically costly, requiring large amounts of ATP. It needs to be stopped to preserve cellular ATP. The large serine/threonine protein kinase mTOR (mechanistic target of rapamycin) is a central regulator of nutrient, energy, and growth factor signaling. It also regulates the activity of the translational machinery, deregulated in most solid tumors. Importantly, *STK11/LKB1*-AMPK activation inhibits the mTOR pathway leading to the inhibition of protein synthesis, thus limiting ATP intake [2]. Inhibition of mTOR also negatively regulates hypoxia-inducible factor 1 α (HIF-1α), a major regulator of genes involved in cellular metabolism and adaptation to hypoxic conditions by promoting angiogenesis [17]. Moreover, *STK11/LKB1* via AMPK promotes autophagy and mitophagy, a mechanism that is initiated under nutrient starvation which increases intracellular metabolic reserves [18,19]. Autophagy is one of the key mechanisms that promotes cell survival in cancer under stress conditions [20].

Besides its role in the cellular metabolism modification secondary to AMPK activation, *STK11/LKB1* has a protective function against active stress. Reactive oxygen species (ROS) are highly reactive molecules produced by aerobic oxygen metabolism. Their accumulation triggers irreversible damages to DNA, especially under glucose starvation or hypoxia [21]. On the one hand, the accumulation of ROS may promote pro-oncogenic mutations and facilitate tumorigenesis; on the other hand, ROS may render cancer cells more vulnerable to cell death. High cellular levels of ROS activate the *STK11/LKB1*-AMPK pathway. It increases the production of NADPH, a key antioxidant, thus decreasing the level of ROS in order to prevent ROS-induced apoptosis [22,23]. Accordingly, *STK11/LKB1* loss enhances levels of intracellular ROS and could lead to an increased sensibility to ROS-induced chemotherapies [24].

To sum up, *STK11/LKB1* is an upstream kinase of the AMPK pathway, a major regulator of cell metabolism under stress conditions (Figure 1). *STK11/LKB1* reschedules cell metabolism by restraining the activity of anabolic enzymes and by promoting the production of instantly available energy with ATP, thus slowing down cell growth and ATP-consuming processes. It also protects the genome from ROS-induced oxidation by regulating antioxidant gene products, and finally promotes cell survival under stress conditions.

## 3. *STK11/LKB1*: A Tumor Suppressor

### 3.1. Molecular Mechanisms of STK11/LKB1 Inactivation in Cancers

*STK11/LKB1* loss of function has been found in several cancer types, mainly through somatic alterations in the *STK11* gene such as non-sense mutation, loss of heterozygosity, insertions, intragenic deletions, or chromosomal deletions [25,26,27,28,29,30,31,32,33]. A recent study of 4446 patients with solid tumors found that the rate of *STK11* alterations was 1.35% (*n* = 60) [34]. Forty-five percent of *STK11*-altered tumors (27/60) were found in NSCLC, 8% in breast, and 7% in head and neck cancer patients. While most mutations affect the kinase domain resulting in loss of kinase activity, others affect production, stability, or localization of the protein [35]. Meanwhile, other non-mutational mechanisms result in an alteration of *STK11/LKB1* expression [36]. *STK11/LKB1* expression can be negatively regulated by hyper-methylation of the *STK11* promoter region as demonstrated in clear cell renal carcinoma [37], colorectal cancer [38], or melanoma [39]. Protein translation can also be reduced at the post-translational level by microRNAs, as shown in cervical [40] and head and neck cancer [41]. These non-mutational mechanisms should be considered in tumor characterization because such tumors could have growth and aggressiveness similar to *STK11*-mutant tumors [36]. Thus, while most clinical studies have used sequencing to characterize *STK11* status, mutation analysis of the *STK11* gene may not be sufficient to identify patients with impaired oncosuppressive *STK11/LKB1* activity [36].

### 3.2. STK11/LKB1 Alterations Promote Lung Cancer Cell Survival and Invasion

**Figure 2:**
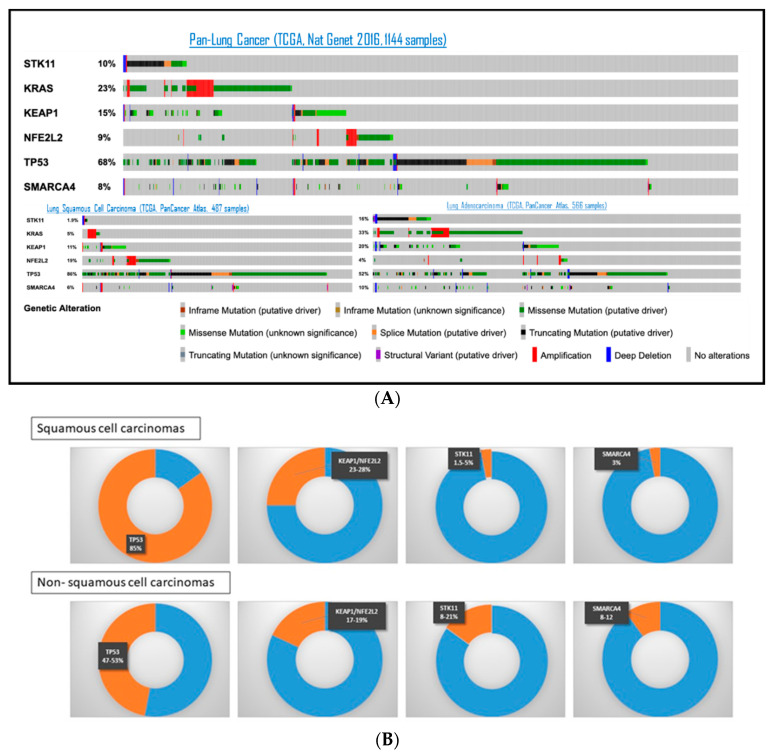
Frequency of most common genomic alterations in tumor suppressor genes among NSCLC patients, based on TCGA (**A**) and the literature (**B**) Panel (**A**) is adapted from https://www.cbioportal.org/ (accessed on 1 October 2021).

The most frequently altered tumor suppressor genes in NSCLC are resumed in Figure 2, based on TCGA (The Cancer Genome Atlas) and data from the literature. *TP53* is the most frequently mutated in both lung adenocarcinoma (LUAD) and squamous-cell carcinomas (SqCC), in 47% and 85% respectively, followed by *KEAP1* or *NFE2L2*, mutated in 17–19% of LUAD and 23–28% of SqCC [42,43]. The prevalence of *STK11* alteration in LUAD is around 8–21% [42,44,45,46,47,48]. In SqCC, *SKT11* alteration was only found in 1.5% to 5% of patients [48,49]. The use of different detection methods between studies (sequencing or immunohistochemistry) could in part explain differences in reported prevalence. Of note, *SMARCA4*, another gene on chromosome 19p, is also found altered at a frequency of 8–12% of NSCLC [50].

Whereas *STK11/LKB1* loss alone was not sufficient to trigger oncogenesis, *STK11* inactivation in a mutant *KRAS*-driven model of mouse lung cancer strongly stimulated growth and metastasis [51]. Latency until tumor growth and metastasis development were shortened compared to the *STK11*-wild-type (WT) counterpart. *STK11*-deficient tumors had increased expression of genes involved in angiogenesis and cell migration, such as NEDD9 (neural precursor cell expressed developmentally down-regulated 9) [51]. In vitro studies showed that *STK11* inactivation increased cell motility, invasiveness, and favored epithelial–mesenchymal transition in lung cancer cells, thus enhancing metastatic potential [52,53]. Moreover, it was shown that *STK11/LKB1* inactivation promotes cancer cell growth and survival via the upregulation of HIF-1α [54]. As explained earlier, inhibition of mTOR by *STK11/LKB1* negatively regulates HIF-1α. Indeed, studies have shown that *STK11/LKB1* inactivation in lung cancer cells led to an upregulated mTOR signaling providing growth advantages [55] associated with mitochondrial dysfunction by autophagy impairment [56]. A mouse model of Peutz-Jeghers syndrome (PJS) with *STK11/LKB1* inactivation showed a dramatic HIF-1α increase via the mTOR pathway [56]. Lastly, mechanisms by which *STK11/LKB1* constrains metastatic ability have been recently studied using LUAD primary tumors (55). Interestingly, *STK11/LKB1* was characterized as a master regulator of chromatin accessibility, through the activation of the transcription factor SOX17.

In addition to its role as an AMPK regulator, *STK11/LKB1* plays a role in glutamine metabolism. Glutamine is a major source of energy in cancer cells and acts as a nitrogen donor in pyrimidine and purine synthesis via the carbamoyl phosphate synthetase 1 (CPS1), a urea cycle enzyme [57,58]. It was demonstrated that *STK11/LKB1* downregulates CPS1 transcription via AMPK-mediated effects and that *KRAS/STK11* mutant lung cancer cells upregulate expression of CPS1, allowing cell division and tumor development [59].

Moreover, glutamine metabolism also plays a crucial role in redox homeostasis via glutathione, as glutathione is implicated in protecting cells from oxidative damage and maintaining redox homeostasis [60]. The enzyme glutamate cysteine ligase condenses glutamate and cysteine in ɣ-glutamylcysteine, then glutathione is produced via glutathione synthetase [61]. *KEAP1* (Kelch-like ECH associated protein 1) is a gene encoding a ubiquitin ligase, a negative regulator of the proteasomal degradation of NRF2 (also known as *NFE2L2*). NRF2 is a transcription factor with a pivotal role in the cellular defense against oxidative stress, by regulating glutathione synthetase [62]. Thus, *KEAP1* promotes transcription of genes encoding detoxifying enzymes and antioxidative stress proteins [43]. As previously mentioned, *STK11/LBK1* loss implies a ROS accumulation.

It has been shown that co-alterations of *STK11/LKB1* and *KEAP1* were significantly associated with lung tumors [49,63,64]. One explanation is the proximity of the corresponding genomic loci on the short arm of chromosome 19 (at 19p13.2 for *KEAP1* and 19p13.3 for *STK11/LKB1*). In *KRAS*-mutated lung cancer, there is a synergic effect of *STK11/LKB1* and *KEAP1* loss: *STK11/LKB1* loss results in an increased level of ROS and high redox stress with an inability to maintain ATP levels. This high redox and energetic stress induces a positive selection for *KEAP1* loss. *KEAP1* loss led to an upregulation of the NRF2 pathway with an increase in glutamate-cysteine ligase transcription catalyzing the production of glutathione. This activation of the NRF2 pathway allows *STK11/LKB1*-deficient cells to detoxify and promote survival in an *STK11/LKB1*-independent way [65].

In conclusion, while *STK11/LKB1* favored cell survival under stress conditions, it has been shown that *STK11/LKB1* loss enhanced cancer cell proliferation under energy deprivation. *STK11/LKB1* deficiency promotes cancer cell growth, motility, and invasion. It also promotes metastasis, through activation of pro-metastatic genes, and regulation of chromatin accessibility.

## 4. *STK11/LKB1* Alterations and Co-Occurring Mutations: Prognostic Impact in Lung Cancer Patients

### 4.1. STK11/LKB1 Co-Occurring Genomic Alterations in Lung Cancer

*KRAS* and *TP53* alterations are present in about half of *STK11*-altered cancers [34,46,48]. *KRAS* mutations are an early mutagenic event in LUAD evolution, as demonstrated by multi-region sequencing that studied *KRAS* mutations in both minimally invasive adenocarcinoma and paired invasive carcinoma [66]. Moreover, 90% of samples with *KRAS* mutations were found to be clonal populations [63]. *KRAS* is the most common proto-oncogene mutated in NSCLC, found in 20–30% of LUAD patients and 5–7% of SqCC patients from Western countries [47,67,68]. Most of *KRAS* mutations occur at codons 12 (86% of patients) and 13 (7% of patients) [47,68]. The most common nucleotide change in metastatic LUAD is from guanine to thymidine (34 G > T or G12C), involving around 40–45% of patients, followed by G12V (15–20% of patients), G12D (16–18% of patients) and G12A (7–10% of patients). Other mutations are observed in less than 5% of patients (G13C, G13D, G12S, G12F, G12R, etc.) [47,67,68,69,70]. It was recently highlighted that *KRAS*-mutant tumors are a very heterogeneous disease, including different tumor subtypes with variable biological backgrounds, different prognoses, and clinical responses to immunotherapy. One hypothesis is that it is due to a greater molecular diversity of tumors defined by mutation subtypes and the presence of co-mutations. Less than 5% of *KRAS*-mutant NSCLC patients have another oncogenic-driven co-mutation, such as *BRAF*, *EGFR*, *PIK3CA*, or *MET* amplification. No concurrent *ALK* or *ROS1* rearrangements have been described [47]. Rare *EGFR* mutations have been described with the *STK11* aberration (1.5% *STK11* mutations occurred in patients with *EGFR* mutations in the pooled analysis from OAK and POPLAR [46,49]). The frequency of *STK11* mutation in each mutational status in NSCLC is reported in Table 1.

*TP53*, *STK11*, and *KEAP1* were the three most commonly co-occurring mutations in *KRAS*-mutant tumors. Of note, the co-mutation patterns of *STK11* and *KEAP1* were similar between *KRAS G12C* mutant vs. *KRAS*-others [63,67]. Nearly half of *KRAS*-mutant patients had *TP53* mutations, followed by *STK11* (18% to 28%) and *KEAP1* (24%) [42,46,47,64,67,69,71]. As previously described, the co-mutation frequency of *STK11* and *KEAP1* significantly co-occurred [49,63,64]. Furthermore, *STK11* and *KEAP1* alterations frequently co-occur in 30% to 60% of *SMARCA4*-mutant NSCLCs [49,72].

The impact of these co-occurring mutations *KRAS/STK11/TP53* has been explored. Patients with NSCLC expressing *KRAS* and *STK11* co-mutations were younger than patients with *KRAS* mutated NSCLC (median age 61 vs. 67 years, *p* = 0.08) [47]. No differences regarding gender, race, smoking history, or performance status were observed. Adenocarcinoma histology and tobacco exposure were documented to be positively associated with *STK11* mutations [73]. Importantly, the distribution of *KRAS*-mutant alleles (G12V, G12C, or G12D) were not related to *STK11* or *TP53* status [64,67]. This suggests that the *KRAS*-specific mutation is not the primary driver of the subsequent mutations. Moreover, clonality analysis from the TCGA dataset found that genetic events such as *STK11*, *TP53*, and *KEAP1* were clonal [64]. Co-occurrence of *STK11* and *TP53* mutations in *KRAS*-mutant tumors was rare in a cohort of untreated, resected LUAD [45]. In a cohort of chemorefractory patients, triple mutant *KRAS/TP53/STK11* tumors were more common [64].

### 4.2. Prognostic Impact of STK11/LKB1 Alterations in Lung Cancer before the Era of Immunotherapy

The impact of *STK11/LKB1* alterations was evaluated in localized, resected NSCLC patients. A retrospective series of 352 surgical NSCLC found that patients harboring mutations in *TP53*, *STK11* or *SMARCA4* had a worse overall survival (OS) after adjustment for confounding factors, with a hazard ratio (HR) for all causes of mortality respectively at 1.47 (95%CI 1.02–2.13), 1.66 (95%CI 1.05–2.61) and 2.1 (95%CI 1.22–3.61) [42]. Amongst patients with *KRAS*-mutant tumors, a significantly worse outcome was seen for the *KRAS/STK11* co-mutant group in comparison to the WT group. These results were validated in a larger cohort from MSK-IMPACT [42]. Conversely, another analysis from the TCGA cohort in the setting of predominantly early-stage surgically resected tumors found that *STK11* alterations were not associated with a decreased OS [64].

Several studies also evaluated the impact of *STK11/LKB1* alterations in advanced NSCLC, before the advent of ICI. In a real-life cohort of advanced NSCLC patients treated with first-line chemotherapy, median OS was shorter in patients with *STK11*-mutant (*n* = 288) vs. *STK11-WT* tumors (*n* = 1849) (11.2 vs. 17.8 months; HR 1.4 (95%CI 1.2–1.6), *p* < 0.0001) [48]. In a smaller cohort of metastatic LUAD patients (17/92 (18%) had *STK11* mutation), *KRAS*/*STK11* co-mutations were associated with worse OS in univariate analyses (HR 2.66; *p* = 0.035) [47]. The *STK11* mutation was found as a negative prognostic factor for OS in patients with either *KRAS* mutation (0.9 years vs. not reached) or *KRAS*-WT (1.46 vs. 2.03 years) [47]. Another retrospective analysis included advanced NSCLC patients who received platinum-based chemotherapy (25/302 (8%) had *STK11* mutation) [46]. No differences in clinical characteristics according to *STK11* status were reported. There was a trend toward shorter survival for the *STK11*-mutant vs. WT groups, with an OS of 10.4 months (95%CI 6.1–15.7) vs. 17.3 months (95%CI 14.0–21.1) (HR 1.53; 95%CI 0.94–2.49; *p* = 0.085), but it was not statistically significant.

Controversies exist regarding the independent prognostic impact of co-occurring mutations in *STK11* with *KRAS*, especially after adjustment for concurrent *KEAP1* and *TP53* mutations and other clinical variables. As previously mentioned, it was demonstrated that *KRAS*/*STK11* tumors have higher rates of *KEAP1* mutational inactivation [64,69]. A large study has evaluated the impact of co-occurring mutations in 330 patients with advanced *KRAS*-mutant NSCLC [69]. The *KRAS*/*STK11* co-mutation was associated with a shorter OS (12 vs. 21 months, HR 1.7; 95%CI 1.1–2.4; *p* = 0.002), but this was not significant after adjustment on major other prognostic factors. In multivariate analysis, only the concurrent mutation in *KEAP1* or *NFE2L2* was independently associated with shorter OS (HR 1.96; 95%CI 1.33–2.92; *p* < 0.001). This study suggested that it was the concurrent *KEAP1* or *NFE2L2* mutation which was associated with a worse prognostic, but not the *SKT11* mutation. There was no impact of a concurrent mutation in *TP53* (HR 0.9; 95%CI 0.6–1.2; *p* = 0.5) [69]. In contrast to *STK11*, *KEAP1* mutations were infrequently observed among *KRAS*/*TP53* co-mutant.

## 5. Immune Impact of *STK11/LKB1* Alterations in Lung Cancer Patients

### 5.1. Tumor-Extrinsic Impact of STK11/LKB1 Alteration: Interaction with Immune System

Recently, there is increasing evidence that loss of *STK11/LKB1* may be involved in the modulation of the tumor immune microenvironment. To understand the underlying mechanisms of the immune surveillance mediated by *STK11/LKB1* alterations, in vivo tumor models were generated with *STK11* gene deletion by CRISPR/Cas9 editing. Genetic ablation of the *STK11* locus in cell lines implanted in syngenic mice resulted in lower numbers of CD3^+^ CD8^+^ and CD3^+^ CD8^+^ /PD1+ T lymphocytes compared to their *STK11*-proficient counterpart, with no impact on CD45^+^ and CD3^+^ CD4 cell infiltrates [71]. Genetic ablation of *STK11* in a *KRAS*-driven murine model of NSCLC demonstrated an accumulation of neutrophils with T-cell suppressive capacities associated with a decreased number of tumor-infiltrating lymphocytes and a reduced expression of PD-L1 in tumors [74]. In an in vitro model, *STK11* extinction did not substantially modify the growth of *KRAS*-mutant colorectal tumors [75]. Conversely, in an immunocompetent mouse model, tumor growth increased, related to a loss of immune control [75]. Kitajima et al. showed that *STK11* loss inactivates the stimulator of interferon genes (STING) expression via DNMT1 and EZH2 epigenetic activity. STING mediates cytosolic DNA-induced signaling events that trigger secretions of type I interferons and diverse chemokines. Without STING, tumor cells cannot detect mitochondrial or nuclear DNA in the cytoplasm leading to a lack of PD-L1 expression and a downregulation of chemokines such as CXCL10 that promote T cell recruitment [76,77]. Studies on cohorts of patients with NSCLC confirmed that *STK11*-mutant tumors could be classified as immunologically ignored, with less immune cell infiltration, as described in the following paragraph [44,45,71].

### 5.2. STK11/LKB1 Alterations Negatively Impact Immune Surveillance in NSCLC Patients

Studies on several cohorts of NSCLC patients retrospectively confirmed preclinical data regarding the impact of *STK11/LKB1* status on the immune microenvironment. In-depth immune profiling was performed on 221 untreated resected LUAD [78]. *STK11*-mutant tumors were characterized by higher neutrophil density, lower CD8^+^ T cells and dendritic cell density, and lower PD-L1 expression. Conversely, *TP53*-mutant tumors were characterized by a higher density of CD8^+^ T cells, indicating a strong adaptive immune response. Moreover, in the *TP53*-mutant subgroup, co-occurring *STK11* mutations were significantly associated with a reduced expression of PD-L1 and a lower CD8^+^ cell density. Importantly, *KRAS* mutations were not implicated in the modulation of the tumor immune microenvironment in this study [78]. Another analysis on resected LUAD found that *STK11* mutations either individually or with *KRAS* mutations were strongly associated with lower NF-κB signature activity, while no such association was seen with *TP53* mutations [45]. Skoulidis et al. focused their analyses on *KRAS*-mutant LUAD and demonstrated that different subsets exhibit distinct patterns of immune system engagement [64]. Tumors with *KRAS*/*TP53* co-mutations had a higher immune system activation, with more T-cell infiltration (such as CD3^+^, CD8^+^, and CD45RO^+^ lymphocytes) and a higher expression of cell-intrinsic co-inhibitory signals such as PD-(L)1. Thus, the presence of *TP53* mutations designated tumors with the strongest adaptive immune response and could potentially allow the identification of patients susceptible to being more sensitive to ICI. Comparatively, *KRAS/STK11* mutated tumors had a lack of immune system engagement. This cold micro-environment could explain a lack of benefit from ICI, as discussed in the following paragraph. *KRAS*-only mutated tumors had a mixed immune microenvironment [64].

Biological markers such as tumor mutation burden (TMB) or PD-L1 expression were demonstrated to be associated with favorable outcomes to PD-1/PD-L1 blockade across diverse tumors [79]. NSCLC patients have among the highest TMB of all malignancies, after melanoma [80]. *KRAS* mutations are associated with an increased PD-L1 expression in NSCLC and are also associated with a high TMB in NSCLC [81,82]. Co-mutant *KRAS*/*TP53* had a higher overall mutation load compared to co-mutant *KRAS*/*STK11* regardless of exposure to tobacco [64]. Moreover, it was recently shown that patients with *STK11* or *KEAP1* alteration had significantly higher blood TMB compared with WT [49,73]. However, *STK11* mutations were associated with a lack of PD-L1 expression in tumor cells, despite the presence of intermediate or high TMB, irrespective of *KRAS* status [71]. While tumor samples with *STK11* mutations were enriched for negative PD-L1 staining [49,73], tumor samples with high PD-L1 expression over 50% were less frequently mutated for *STK11* or *KEAP1* [67,83].

**Figure 3:**
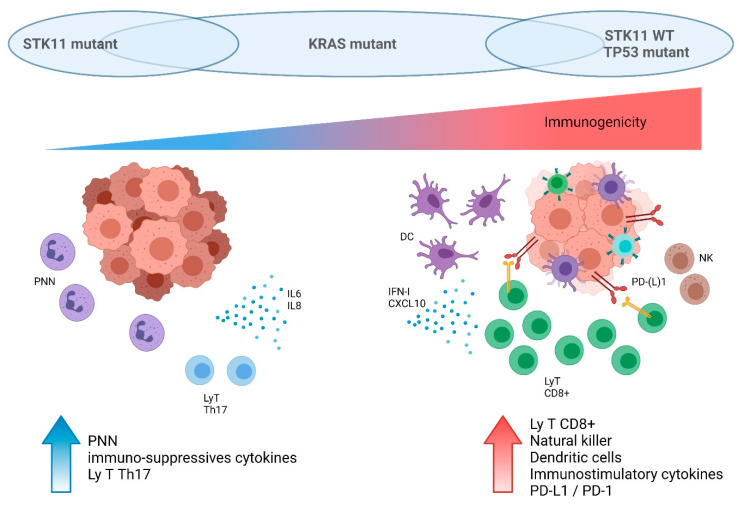
Schematic impact of *KRAS*, *STK11*, and *TP53* status on immune cell tumor microenvironment in lung cancer. This figure was created with BioRender.com (accessed on 1 October 2021).

In conclusion, *STK11*-mutant tumors were associated with T-cell exclusion, low PD-(L)1 levels, and higher density of immune suppressive cells. Thus, these tumors were mostly classified as immunologically ignored, or “cold” tumors, as resumed in Figure 3. The impact of this poor immune surveillance was then studied in NSCLC patients treated with ICI.

### 5.3. Impact of SKT11/LKB1 Alterations on ICI Efficacy in NSCLC Patients

ICI-based therapies are the new standard of care in NSCLC patients without oncogenic addiction, in monotherapy, or in combination with chemotherapy [3]. ICI demonstrated around a 30% reduction in the risk of death compared with chemotherapy for advanced NSCLC [84]. However, around half of the pre-treated patients did not have any benefit from ICI and developed early progression [85,86]. The impact of mutations such as *KRAS* and co-occurring mutations on disease behavior and clinical outcomes is an important matter for debate. Therefore, whether *STK11* genomic alterations may predict a lack of clinical benefit from ICI was further investigated. In vivo studies showed that a syngenic mice model implanted with *STK11* knockout cells was resistant to PD-1 blockade, contrary to their *STK11*-proficient counterpart [71,75]. Whereas cell lines with *STK11*-deficiency result in a higher TMB, co-occurring *KRAS/STK11* mutations in a mouse model decreased the efficacy of anti-PD-1 therapy compared to the *KRAS/TP53* co-mutant model [87]. This resistance to ICI was confirmed in different tumor cell types (colorectal CT26 *KRAS*-mutant or EMT6 mouse mammary *KRAS*-WT) [75].

In patients, several retrospective studies have evaluated the prognostic or predictive impact of *STK11/LKB1* status on ICI efficacy (Table 2).

A cohort from the MSK-IMPACT included 240 NSCLC patients treated with anti-PD1 therapy alone or in combination with anti-CTLA4 and evaluated the impact of gene alterations identified by NGS on ICI efficacy [88]. Like other cohorts, *SKT11* was the most enriched altered gene in PD-L1 negative tumors, but this association was not statistically significant. *STK11* alterations were associated with a lack of benefit from ICI, mostly in pre-treated patients. Another study found that within a small cohort of 66 previously treated patients with a PD-L1 ≥ 1% receiving ICI, *STK11*-mutant tumors had significantly lower response rates vs. *STK11*-WT (0% vs. 34.5%, *p* = 0.026) regardless of *KRAS* status [71]. *STK11* mutations were also associated with a shorter PFS (HR 4.76, 95%CI 2.0–11.1, *p* = 0.00012) and OS (HR 14.3, 95%CI 3.4–50.0, *p* < 0.0001) with ICI treatment [71].

However, studies of other cohorts found controversial results on the prognostic and/or predictive role of *STK11*. A study conducted among 86 *KRAS*-mutant NSCLC patients who received ICI concluded that neither the presence of *TP53* nor *SKT11* was associated with a difference in OS from the start of ICI [69]. Importantly, patients with a co-occurring mutation in *KEAP1* or *NFE2L2* were found to have a shorter OS in both univariate and multivariate analysis (HR 3.54, 95%CI 1.55–8.11, *p* = 0.003). Several larger cohorts compared ICI and also chemotherapy efficacy according to *STK11* status. A real-world study including 2276 previously untreated patients demonstrated that *STK11*/*KEAP1* mutations are more prognostic than predictive biomarkers of resistance to ICI [73]. Indeed, these mutations conferred a poor prognosis regardless of treatment class (ICI, chemotherapy, targeted therapies, or antiangiogenic therapies). Another large real-life cohort has evaluated the impact of *STK11* status on first-line treatment efficacy, including 270 patients receiving first-line ICI-based therapy, and 770 patients ICI treated in the latter line [48]. There was a trend for a shorter OS in first-line therapy for patients with the *STK11*-mutant (*n* = 40) compared with *STK11*-WT tumors (*n* = 230) (11.2 vs. 17.7 months; HR 1.4 (95%CI 0.9–2.3), but it was more pronounced in the second-line ICI group for *STK11*-mutant (*n* = 211) vs. *STK11*-WT patients (*n* = 559) (6.3 vs. 12.0 months; HR 1.6 (95%CI 1.3–2.0). Finally, a pooled analysis from OAK and POPLAR studies showed that among patients receiving atezolizumab in second or third-line settings, those with *STK11/KEAP1* tumor mutation had worse median OS compared with WT patients (7.3 vs. 15.6 months (*p* = 0.004)) [49]. Survival analysis among NSCLC patients harboring *STK11* alterations demonstrated that atezolizumab did not improve OS compared to docetaxel, suggesting resistance to ICI treatment among *STK11*-mutant. However, importantly, this was also observed among patients receiving docetaxel. Thus, in these studies, *STK11* mutations did not show different treatment-specific effects between atezolizumab and docetaxel.

Recently, the impact of *STK11* or *KEAP1* status at diagnosis on immune resistance during first-line therapy has been investigated. Exploratory analyses from two phase 3 trials were presented during AACR 2020 [89,90]. The first study, KEYNOTE-189, evaluated chemotherapy +/− pembrolizumab or placebo as first-line therapy for non-squamous NSCLC. The other study was KEYNOTE-042, which evaluated pembrolizumab monotherapy vs. chemotherapy as first-line for PD-L1-positive NSCLC. Whole-exome sequencing data from tumor and normal DNA was available for 47% and 34% of patients, respectively.

**Figure 4:**
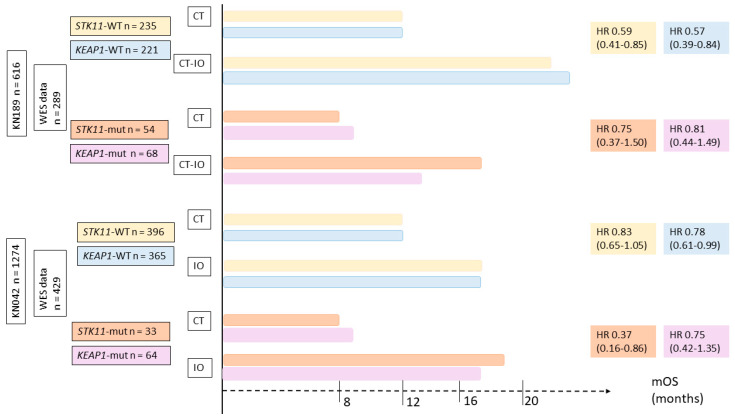
Impact of *STK11* or *KEAP1* status on immune resistance during first-line therapy based on two phase III trials, evaluated chemotherapy versus immunotherapy (KEYNOTE 042) or versus chemo-immunotherapy (KEYNOTE 189). CT = chemotherapy, IO = immunotherapy.

For both studies, monotherapy with ICI or in combination with chemotherapy was associated with better outcomes than chemotherapy alone, regardless of *STK11* or *KEAP1* status. Results on OS by treatment and mutation type are summarized in Figure 4.

In conclusion, *STK11* status was not identified as a predictive biomarker of resistance to ICI, but more as a negative prognostic factor regardless of treatment type. These exploratory analyses strongly support the use of ICI (alone or in combination) in first-line settings for NSCLC patients, regardless of *STK11,* or *KEAP1,* status. These results highlighted the fact that each individual mutation is insufficient to predict ICI response or resistance.

## 6. Conclusions

**Figure 5:**
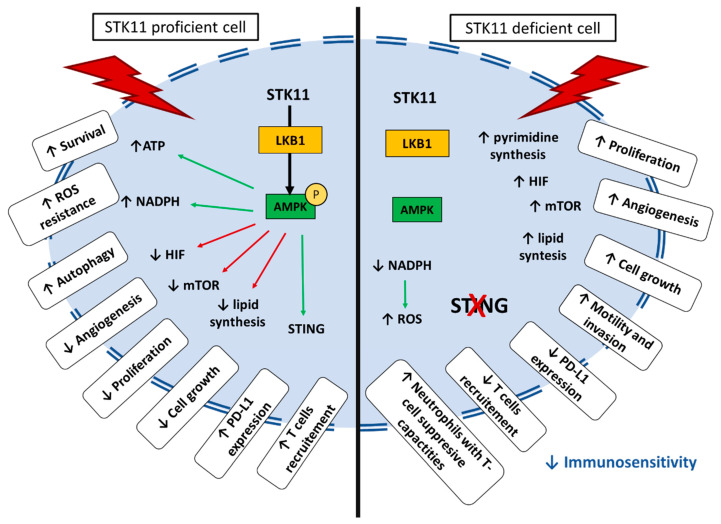
Schematic representation of impact of *STK11*-proficient or deficient cell under stress condition.

*STK11/LKB1* acts as a major regulator of energy sensing and cellular metabolism. It activates the canonical AMPK pathway, a well-described central metabolic sensor that regulates lipid and glucose metabolisms. Intracellular signaling downstream of *STK11/LKB1* also regulates the mTOR pathway to modulate protein synthesis and cellular proliferation. Moreover, *STK11/LKB1* has been reported to have tumor suppressor activity. Inactivating somatic mutations of *STK11* have been observed in different types of solid tumors, especially in NSCLC, where it modulates cancer cell differentiation, invasion, and metastasis. *STK11* mutations in tumor cells also have tumor-extrinsic functions. Indeed, *STK11*-mutant tumors were associated with low PD-(L)1 levels, cytotoxic T-cell exclusion, and a higher density of the immuno-suppressive cell population. The biological function of *STK11/LKB1* and the impact of its loss under stress conditions are resumed in Figure 5.

Despite sharing common clinical-pathological factors, NSCLC patients have distinct phenotypes linked to a unique tumor microenvironment that regulates tumor behavior and response to therapies. Thus, alterations in the *STK11* gene in NSCLC have recently emerged as an important regulator of immune response and a potential resistance biomarker to ICI. However, the effect of *STK11/LKB1* inactivation on clinical response to PD-(L)1 is a subject of much debate. While some authors suggest that *STK11/LKB1* inactivation could favor primary resistance to ICI, several questions have emerged. Are *STK11/LKB1* alterations really a predictive biomarker, or only a prognostic one? Are they confounding factors due to co-occurring mutations, such as *KEAP1*? The clinical and biological significance of associated genetic events is unclear. Will physicians have to adapt their therapeutic choices in the future based on *STK11/LKB1* tumoral status?

So far, guidelines do not recommend to systematically characterize *STK11/LKB1* status as it has not been demonstrated to impact therapeutic strategy. It is important to highlight the fact that exploratory analyses support the use of ICI (alone or in combination) in first-line settings for all NSCLC patients without oncogenic addiction, regardless of *STK11* or *KEAP1* status when available. However, the impact of tumor suppressor genes such as *STK11, KEAP1*, and *TP53* co-mutations may help refine response prediction algorithms in both PD-L1 positive and negative tumors. Whether it could impact the decision to add chemotherapy to ICI in high-PDL1 patients is still unknown.

## Figures and Tables

**Table 1:** Frequency of STK11 mutation in each mutational status in NSCLC. TCGA. Nat Genet 2016. 1144 samples. HD: homozygous deletion) from https://www.cbioportal.org/ (accessed on 1 October 2021).

Mutational Status	Total Number of Mutated Patients	Number of Patients with Co-Mutation Gene/STK11	% Co-Mutation
KRAS	259	63	24.32
NTRK1	96	18	18.75
NRAS	30	4	13.33
SMARCA4	94	11	11.70
BRAF	80	9	11.25
NKX2-1	99	11	11.11
ALK	57	5	8.77
ROS1	71	6	8.45
CDKN2A (HD)	342	25	7.29
TP53	776	50	6.44
RET	48	3	6.25
MAP2K1	19	1	5.26
EGFR	164	7	4.27
ERBB2	50	2	4.00
MET	57	2	3.51
PIK3CA	276	9	3.26

**Table 2:** Major retrospective studies evaluating prognostic or predictive impact of *STK11* status on ICI efficacy.

Publication	Population	Number of Patients	Impact of *STK11* Mutation on OS	Conclusion
Rivizi J Clin Oncol 2018	MSK-IMPACT Cohort - Advanced NSCLC - ICI monotherapy or ICI combination - Mostly latter lines of treatment	*n* = 240 received ICI Comparison to a non-ICI group (*n* = 608)	*STK11*-mutant tumors were significantly enriched in the no durable benefit group (progressive disease or stable disease shorter than 6 moths), compared to the non-ICI group	Alterations in *STK11* were associated with lack of benefit from ICI
Skoulidis Cancer Discov 2018	MDACC cohort - Advanced NSCLC - ICI monotherapy - PD-L1 ≥ 1%	*n* = 66 All patients received ICI (no other groups)	*STK11* mutations were associated with shorter OS - HR 14.3, 95% CI 3.4–50.0, *p* < 0.0001	Alterations in *STK11* were associated with lack of benefit from ICI
Papillon-Cavanagh ESMO Open 2020	Retrospective real-world cohort - advanced NSCLC - first-line ICI, chemotherapy, or targeted therapies	*n*= 2276 574 (25%) received first-line ICI-based therapies	*STK11* mutations were associated with shorter OS - HR 1.57 [1.13–2.19] in the first-line ICI group - HR 1.44 [1.15–1.8] in the first-line chemotherapy group	*STK11* confers a poor prognosis, regardless of treatment class with ICI or chemotherapy or targeted therapies
Shire Plos One 2020	Retrospective real-word cohort - advanced NSCLC - first-line ICI-based therapies or chemotherapy	*n* = 2407 patients 270 (11%) received first-line ICI-based therapies	Median OS was shorter for patients with *STK11*-mut compared with patients with *STK11 WT*: - In the first-line IO group, 11.2 vs. 17.7 months, HR, 1.4 [95% CI, 0.9–2.3] - In the second-line IO group, 6.3 vs. 12.0 months, HR, 1.6 [95% CI, 1.3–2.0] - In the first-line chemotherapy group, 11.2 vs. 17.8 months, HR, 1.4 [95% CI, 1.2–1.6]	*STK11* confers a poor prognosis, regardless of treatment class with ICI or chemotherapy
Shang Lung Cancer 2021	Selected population from the POPLAR and OAK studies - Advanced non-squamous NSCLC - All received one or two prior systemic therapies	*n* = 598 304 received atezolizumab 294 received docetaxel	Median OS was shorter for patients with *STK11*-mut compared with patients with *STK11 WT*: - In the atezolizumab group, 7.3 vs. 15.6 months (*p* = 0.004), HR = 0.623; 95 %CI: 0.408−0.951; *p* = 0.028 - in docetaxel cohort, HR = 0.626; 95 %CI: 0.407−0.962; *p* = 0.033.	*STK11* confers a poor prognosis, regardless of treatment class with ICI (atezolizumab) or chemotherapy (docetaxel)

## Data Availability

Not applicable.
